# Low-level laser treatment’s ability to reduce dry socket pain

**DOI:** 10.2340/aos.v83.42261

**Published:** 2024-11-12

**Authors:** Giuseppe Minervini, Rocco Franco, Mirko Martelli, Salah Hafedh, Maria Maddalena Marrapodi, Marco Di Blasio, Patrizio Bollero, Marco Cicciù

**Affiliations:** a Saveetha Dental College and Hospitals, Saveetha Institute of Medical and Technical Sciences (SIMATS), Saveetha University, Chennai, India; b Multidisciplinary Department of Medical-Surgical and Odontostomatological Specialties, University of Campania “Luigi Vanvitelli”, Naples, Italy; c Department of Biomedicine and Prevention, University of Rome “Tor Vergata”, Rome, Italy; d Department of Clinical Sciences and Translational Medicine, University of Rome “Tor Vergata”, Rome, Italy; e Orthodontics Department, Faculty of Dentistry, Sana’a University, Sana’a, Yemen; f Department of Woman, Child and General and Specialist Surgery, University of Campania “Luigi Vanvitelli”, Naples, Italy; g University Center of Dentistry, Department of Medicine and Surgery, University of Parma, Parma, Italy; h Department of System Medicine, University of Rome Tor Vergata, Rome, Italy; i Department of Biomedical and Surgical and Biomedical Sciences, Catania University, Catania, Italy

**Keywords:** Dry socket, laser therapy, alveolar osteitis

## Abstract

After a tooth extraction, a dry socket is a common problem that can cause excruciating pain and prevent healing. Antiseptic dressings have historically been the mainstay of treatments for this illness in order to lower bacteria and accelerate healing. Alveogyl is a medicated dressing composed of butamben, iodoform, and eugenol is conventionally used to manage the pain associated with dry socket. The purpose of this study is to assess how well laser therapy treats alveolitis symptoms. The idea that laser therapy is an excellent tool for treating alveolitis is what motivated this meta-analysis. This systematic review aims to evaluate the effects of Level Laser Therapy (LLT) in the treatment of dry socket.

**Methods:**

A literature search was done on PubMed, Lilacs, Web of Science, Scopus, and Cochrane using the keywords entered, and papers published between January 2000 and September 2023 were taken into consideration. The terms “laser” and “dry socket” have been merged using the Boolean conjunction AND; the results show that 65 studies could be identified using the three search engines. Only five were selected to create the current systematic study and metanalysis. The meta-analysis demonstrated that laser therapy is superior to the traditional Alvogyl treatment in managing alveolitis symptoms, especially in pain reduction. The overall effect demonstrated a mean difference of −2.01 (95% CI: −2.43 to −1.59) on the third day of treatment, with a *p* < 0.05, indicating statistical significance.

**Conclusion:**

The quantitative analysis showed that Low-Level Laser Therapy demonstrated promising potential in managing alveolitis symptoms, particularly in terms of pain reduction, when compared to traditional treatments like Alvogyl. Despite the results indicating a statistically significant reduction in pain, the evidence does not conclusively establish laser therapy as a complete substitute for conventional therapies. Further high-quality studies with larger sample sizes and standardized protocols are required to confirm its long-term efficacy and to assess its broader applicability in clinical settings.

## Introduction

Dry socket (DS) is a common complication after tooth extraction, causing severe pain and impeding healing. Treatments addressing this condition have traditionally relied on antiseptic dressings to reduce bacteria and promote healing. Recent research suggests that laser treatment, a minimally invasive procedure, may offer an effective alternative to conventional treatments for dry sockets. Tooth extraction is a standard dental procedure but can result in a postoperative complication known as a dry socket. Dry socket occurs when the blood clot that usually forms after an extraction is lost prematurely [1]. This condition can lead to severe pain, delayed healing, and infection. Additionally, alveolitis also predisposes the area to bacterial contamination, resulting in secondary infections and further complications. Various risk factors contribute to the development of alveolitis, including smoking, poor oral hygiene, oral contraceptive use, and traumatic extractions. Smoking, particularly, has a well-established association with the condition, as nicotine causes vasoconstriction, limiting blood flow to the healing tissues, and disrupting clot formation. Additionally, aggressive mouth rinsing, drinking through straws, and any activity that generates suction in the mouth might dislodge the blood clot, increasing the likelihood of dry socket formation [2, 3]. Clinically, alveolitis is diagnosed based on symptoms such as severe pain, the absence of a visible blood clot in the socket, and a foul odor or taste from the exposed bone. Conventional treatments for dry sockets include using antiseptic dressings, such as those containing [4–8] chlorhexidine and eugenol. There is disagreement in the indexed literature regarding the most exact therapeutic protocol for managing dry sockets. Traditionally, DS is treated with a paste called zinc oxide, composed of a zinc oxide and eugenol combination [9–14]. Other pharmacologic preparations and platelet-rich fibrin have been proposed to significantly help in reducing post. These dressings reduce bacterial load and promote healing [15]. However, they can be uncomfortable and require multiple applications. Recently, laser treatment has been proposed as an alternative treatment for dry sockets [16]. This treatment uses low-level lasers to reduce inflammation, promote wound healing, and reduce pain. It is a minimally invasive procedure that is relatively painless and can be completed in a single session [17]. A recent study compared the effectiveness of laser treatment and antiseptic dressings in treating dry sockets [18]. Previous studies and systematic literature reviews have evaluated the different therapies of DS [18–26]. Laser therapy has gained popularity in various dental applications, including soft and hard tissue procedures. Low-level laser therapy (LLLT) and high-level laser therapy (HLLT) have demonstrated beneficial effects on wound healing, inflammation reduction, and pain management.

Recent studies have explored the potential of laser therapy as an adjunct or alternative treatment for dry socket. LLLT has been shown to stimulate cellular activity, promote angiogenesis, and modulate the inflammatory response, which may accelerate healing and alleviate pain associated with dry socket. HLLT, with its precision and ability to target specific tissues, offers a non-invasive and efficient option for managing dry socket.

The study included 60 patients who had undergone tooth extraction and were diagnosed with dry sockets. Half of the patients were treated with antiseptic dressings, and the other half were treated with laser therapy. The results showed that the laser treatment group had significantly less pain and a faster healing rate than the antiseptic dressing group. They also had fewer side effects, such as infection and inflammation. LLLT has recently become well known among treatment methods for various medical issues, including wound healing, musculoskeletal issues, and pain management. It has been discovered that LLLT has a beneficial overall impact on the inflammatory processes and speeds up and improves the quality of wound healing [19]. When applied to oral mucosa, it has also demonstrated the ability to be antimicrobial [27–36]. The SaliCept patch (Carrington Laboratory, Irving, TX) is a more recent treatment option. It is a freeze-dried preparation of acemannan hydrogel, a mixture of naturally occurring substances with acemannan as its main component. Acemannan is a -(1,4)-acetylated mannan obtained from the transparent inner gel of Aloe vera. The importance of the topic for clinicians led to the analysis of this topic. The occurrence of alveolitis is around 20% for third molar extraction. Therefore, no meta-analyses and reviews in the literature specifically address LLLT to resolve painful symptoms. Therefore, we performed this systematic literature review with meta-analysis. This review aims to evaluate the effects of laser therapy on post-extraction alveolitis, a common complication of surgical extractions. The hypothesis of our systematic review and meta-analysis is to evaluate the efficacy of laser therapy as an adjuvant for treating DR. The laser hypothesis is a reliever of post-extractive alveolitis pain.

## Materials and methods

### Eligibility criteria

Based on the following Population, Exposure, Comparator, and Outcomes (PICO) model, we evaluated each document for eligibility:

(P) Participants: patients who have undergone extraction and suffer from dry sockets.

(I) Exposure: patients with DS treated with laser therapy.

(C) Comparison: patients with DS treated with different types of therapy.

(O) Outcome: the first outcome is to evaluate the effectiveness of LLLT on reducing pain and discomfort in DS patients. A primary purpose is to evaluate the efficacy of laser treatment on patients with DS, and a secondary purpose is to evaluate its preventive efficacy.

The following inclusion criteria were used: (1) articles in English, (2) human studies, (3) clinical trials, and (4) randomized clinical trials.

The following were listed as exclusion criteria: (1) non-PICO articles, (2) duplicate articles, (3) books, (4) letters to editors and experimental studies;, (5) review articles, (6) case series, (7) case report, and (8) patients with systemic disease.

### Search strategy

We searched PubMed, Web of Science, Lilacs, Scopus, and Cochrane databases for publications published between January 2000 and September 2023, using a systematic search strategy. Manual searches were also done on the same topic-related systematic reviews from the past. Two reviewers (GM-RF) were involved in the literature search during the process of screening and reading abstracts. Where there were discrepancies in the inclusion of articles, they were resolved by a third reviewer (ALG). The MeSh phrases were utilized in PubMed, but a manual search was done to make up for their absence in the other search engines. The study was registered in the PROSPERO database under number CRD 4453468737 (Table 1).

**Table 1:** Search strategy.

***PubMed***
(“dry socket”[MeSH Terms] OR (“dry”[All Fields] AND “socket”[All Fields]) OR “dry socket”[All Fields]) AND (“laser s”[All Fields] OR “lasers”[MeSH Terms] OR “lasers”[All Fields] OR “laser”[All Fields] OR “lasered”[All Fields] OR “lasering”[All Fields])
***Web of Science***
((ALL=(dry socket)) AND ((ALL=(laser))
***Lilacs***
“dry socket”(palavras) AND “laser”(palavras)
***Scopus***
TITLE-ABS-KEY (low AND light AND laser AND therapy AND dry AND socket)
***Cochrane***
TITLE-ABS-KEY (low AND light AND laser AND therapy AND dry AND socket)

### Data extraction

The information was gathered from the included papers by two reviewers (AR) and (RF) independently utilizing a tailored data extraction on an Excel spreadsheet. If there were any differences of opinion, a third reviewer helped to reach a consensus (R.F.).

The following information was taken out: (1) First author, (2) Year of publication, (3) Sample, (4) Therapy Type, (5) Pain Assessment, and (6) Therapy Results. Table 2 now contains the data that were taken out and added. The publications were all read by two authors independently, and the data were compared and placed in their proper context in the table.

**Table 2:** Main characteristics of the studies included in the present systematic review.

Author	Year	Sample	Type of Therapy	Evaluation of pain	VAS control vs study	Results of therapy
ALHarthi et al.	2023	55 Patients:	Group 1: MC	VAS scale	VAS study: 2.07	LLLT reduce pain concerning conventional therapy
		Group 1: 14	Group 2: MC with alveogyl		VAS control: 1.14	
		Group 2: 13	Group 3: Alveogyl with PBMT			
		Group 3: 14	Group 4: PBMT			
		Group 4: 14				
Eshghpour et al.	2015	60 Patients:	Group 1: alveogyl	VAS scale	VAS study: 0.3	LLLT reduce pain concerning conventional therapy
		Group 1: 20	Group 2: LPRL		VAS control: 0.5	
		Group 2: 20	Group 3: LPIL			
		Group 3: 20				
Kaya et al.	2011	104 Patients:	Group 1: curettage	VAS scale	VAS study: 0.3	LLLT reduce pain concerning conventional therapy
		Group 1: 26	Group 2: MC with alveogyl		VAS control: 3	
		Group 2: 26	Group 3: MC with salicept			
		Group 3: 26	Group 4: MC with LLLT			
		Group 4: 26				
Rani et al.	2016	60 patients:	Group 1: alveogyl	VAS scale	VAS study: 1.5	LLLT reduce pain concerning conventional therapy
		Group 1: 20	Group 2: LLLT		VAS control: 3.3	
		Group 2: 20	Group 3: Erbium Laser			
		Group 3: 20				
Kamal et al.	2020	45 patients:	Group 1: MC and alveogyl	VAS Scale	VAS study: 1	LLLT reduce pain concerning conventional therapy
		Group 1: 30	Group 2: LLLT		VAS control: 4	
		Group 2: 15				

LLLT: Low-Level Laser Therapy; VAS: Visual Analogue Scale; PBMT: Photobiomodulation Therapy; MC: Mechanical Curettage.

### Quality assessment

Two reviewers (GM and RF) assessed the risk of bias using Version 2 of the Cochrane risk-of-bias tool for randomized trials (RoB 2). Any disagreement was discussed until a consensus was reached with a third reviewer (AC).

### Statistical analysis

The pooled analyses were performed using Review Manager version 5.2.8 (Cochrane Collaboration, Copenhagen, Denmark; 2014). Algogyn therapies in combination with curettage were compared with the use of laser in the treatment of DS. Inverse variance with random effects was used to compare different therapies. The risk ratio between the two groups was measured. Heterogeneity among studies was evaluated using the Higgins Index (I2) and the chi-square test and classified as follows: low heterogeneity (< 30%), medium heterogeneity (30% – 60%), and high heterogeneity (> 60%).

## Results

### Study characteristics

Sixty-five studies were located after the study, due to the search done using the three engines. 13 items were disqualified at the initial phase due to duplicates. In contrast, five were disqualified due to language barriers. Twenty-seven articles were removed from both search engines during the initial screening phase because they were systematic literature reviews, so they did not fit the inclusion requirements. Additionally, a filter was added that only considered randomized clinical trials. The abstracts of five publications were assessed during the final screening process.

**Figure 1:**
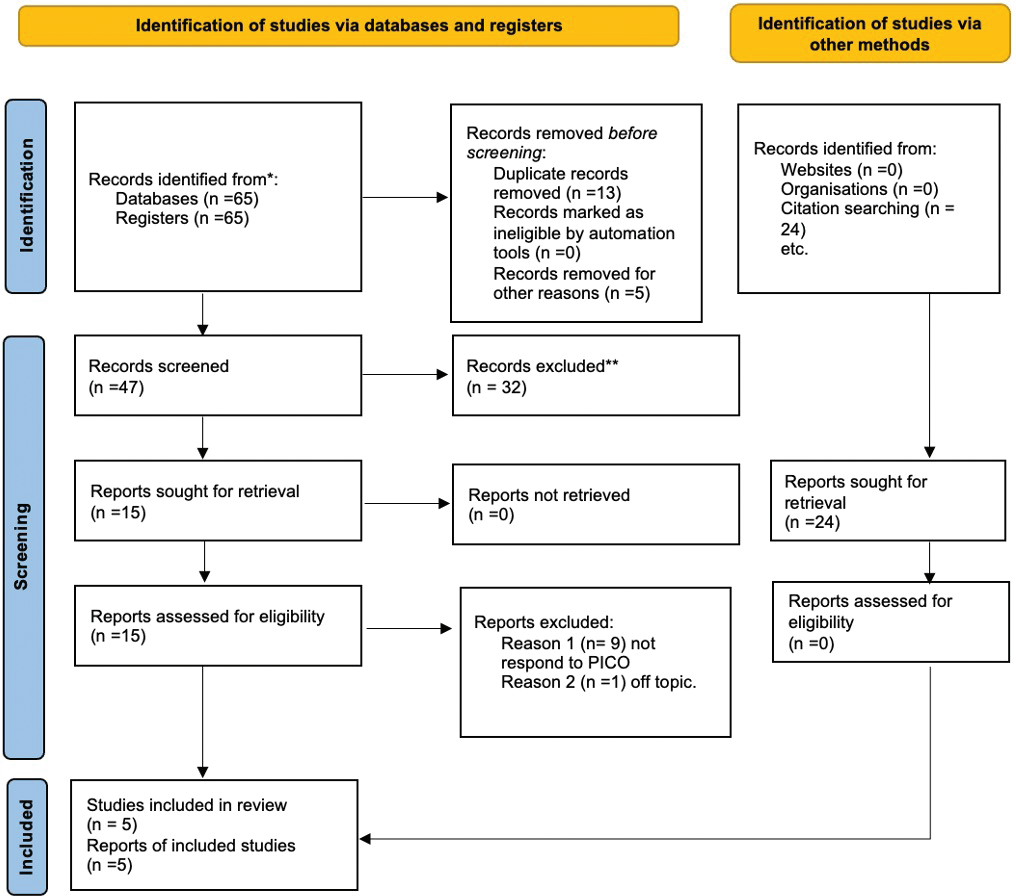
Prisma flowchart.

Only five papers were picked to create the present systematic study, as illustrated by the Preferred Reporting Items for Systematic Reviews and Meta-Analyses (PRISMA) 2020 flowchart in Figure 1; 10 articles were excluded: nine did not meet Population, Exposure, Comparator, Outcome (PECO), one article dealt with the incidence of DS, and one reported alternative techniques to treat DS and didn’t have a control group. According to the PECO model, papers were selected for the title and abstract screening. Finally, five articles were present in the publication on the search engines used. Alternatively, a manual search was performed from the bibliography and websites. Fifteen articles were selected, and the abstract was read. However, they were excluded because they did not meet the PECO model, and some were duplicates. The remaining articles were selected and screened for the title and abstract screening according to the PECO model. The studies considered have a time frame from 2011 to 2023. Abstracts were read, and groups that compared alvogyl and laser were selected and considered. The other groups with mechanical therapy were excluded from the meta-analysis. The studies in this meta-analysis do not consider laser power and setting. Therefore, the studies have much heterogeneity. All studies assess post-alveolate pain with the VAS scale. The studies analysed were conducted in various parts of the world: Arabia, Iran, the USA, and India. A total of 205 subjects were analyzed. Of these patients, 96 patients belong to the study group in which the efficacy of the laser is evaluated, and 109 patients belong to the control group in which they were treated with Algogyn. All studies are RCTs and therefore have two or more groups. The pain scale was submitted to patients after 3 days in all studies to ensure the homogeneity of the studies.

### Main findings

The study of AlHarthi evaluates the difference in pain between alvogyl and laser. No studies compare how Alveogyl and adjunct photobiomodulation therapy (PBMT) affect individuals with alveolar osteitis’ self-rated postoperative pain (SPP) (dry socket). The current randomized controlled trial’s objective was to determine how well Alveogyl with and without PBMT managed SPP in individuals with DS. Included were adult nonsmokers with a diagnosis of DS. Four sets of patients were randomly assigned. Patients in Group 1 experienced mechanical curettage (MC) with generous irrigations of normal saline. Patients in Group 2 had MC + Alveogyl dressings applied to their extraction sites, which were changed every 48 h until their discomfort subsided. A diode laser was used to perform MC + Alveogyl on the subjects in Group 3. Patients in Group 4 received only PBMT therapy. SPP was evaluated using the visual analogue scale (VAS) up to 3 and 6 days after. Using logistic regression models, the relationship between SPP scores and age, sex, and tooth eruption state was evaluated. *P*-values 0.01 and higher were considered statistically significant. There were a total of 14, 13, 14, and 14 DS patients in groups 1, 2, 3, and 4, correspondingly. All of the patients had their mandibular third teeth extracted. SPP in all categories remained the same at baseline and on day 1. On days 2 and 3, Group 2 had substantially higher mean VAS scores than Group 3 at the T1 and T2 intervals (*P* 0.01 and *P* 0.01, respectively). On days 2 and 3, Group 4 had substantially higher mean VAS scores than Group 3 at the T1 and T2 intervals (*P* 0.01 and *P* 0.01, respectively). At the T0 and T1 intervals on day 3, there was no change in SPP between groups 3 and 4 [37].

Kaya’s randomized prospective clinical trial was to evaluate the effectiveness of LLLT, the SaliCept patch, and alvogyl in the treatment of alveolar osteitis. One hundred four patients referred to our clinic with an alveolar osteitis complaint made up the study group. The patients were divided into four groups at random: group 1 received only curettage and irrigation; Group 2 underwent curettage and irrigation followed by the direct application of alvogyl; Group 3 underwent curettage and irrigation followed by the direct application of a SaliCept patch; and group 4 underwent curettage and irrigation followed by diode laser treatment. The therapy procedures were repeated 3 days later. Clinical signs and symptoms were recorded for each patient at diagnosis, 3 days later, and 7 days later. Between groups 2 and 3, there were no statistically significant variations in the treatment of alveolar osteitis. However, group 4 significantly outperformed the other three groups in managing alveolar osteitis [38].

Eshghpour’s research examined the effectiveness of LLLT for treating alveolar osteitis. Three groups of 60 individuals with mandibular third molar alveolar osteitis were randomly created. In group 1, alvogyl was inserted after socket irrigation, and the procedure was done 48 h later. A low-power red laser irradiated the receptacle in group 2 for 3 days. The participants in Group 3 received the same low-power infrared laser therapy as those in Group 2 with the same set of guidelines. The pain level was measured for 3 days using a VAS in the morning (T0, before intervention) and 6, 11, and 12 h afterwards. At T1 and T2 on day 1 and at T0 and T1 on day 2, the alvogyl group experienced significantly less pain than the other groups (*p* 0.05). At the T2 point on days 2 and 3, the red laser group’s VAS substantially decreased compared to the other groups (*p* 0.05). At any therapy intervals, the infrared laser was no more effective than the other groups, but it did lower VAS to a tolerable level [39].

Rani’s randomized trial evaluated the efficacy of laser in the treatment of alveolitis. It randomized patients into three different groups. In group 1, patients were treated with alvogyl, in group 2 with a diode laser, and in group 3 with an erbium laser. VAS scale and healing after 7 days were evaluated. There was better pain control in groups II and III than in group I. The difference was statistically significant between groups II, I, and III (*p* 0.05). The pain control in group III was better than group I [40].

Kamal’s study in India evaluated 45 patients, randomly divided according to treatment. In Group 2, patients received conventional therapy with Alvogyl, while in Group 2, with LLLT. Patients were assessed according to pain using the VAS scale at 0, 4, 7 days after dry socket treatment. The results showed that in laser therapy, in the conventional treatment group I, the pain score was 7–10 on the day of presentation (day 0) and the pain score dropped to 4–6 on day 4 and further decreased to 2–4 on day 7; however, in the study group patients who received LLLT, a similar pain score of 7–10 was recorded on the day of presentation (day 0), and the pain score dropped to 1–2 on day 4 and further improved to 0–1 on day 7 [18].

### Meta-analysis

**Figure 2:**
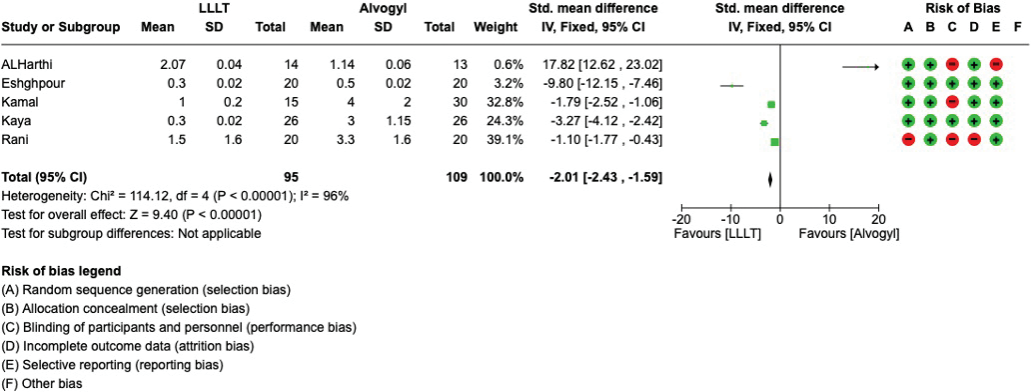
Forest plot of the meta-analysis.

The meta-analysis was conducted by random model effect because of the high heterogeneity (*I*^2^ = 96%) between the five included studies. The overall effect, reported in Figure 2, the Forest plot found that laser therapy has a higher efficacy on the third day on pain (mean difference –2.01; 95% CI from −2.43 to −1.59) with a *p* < 0.05.

### Quality assessment and risk of bias

**Figure 3:**
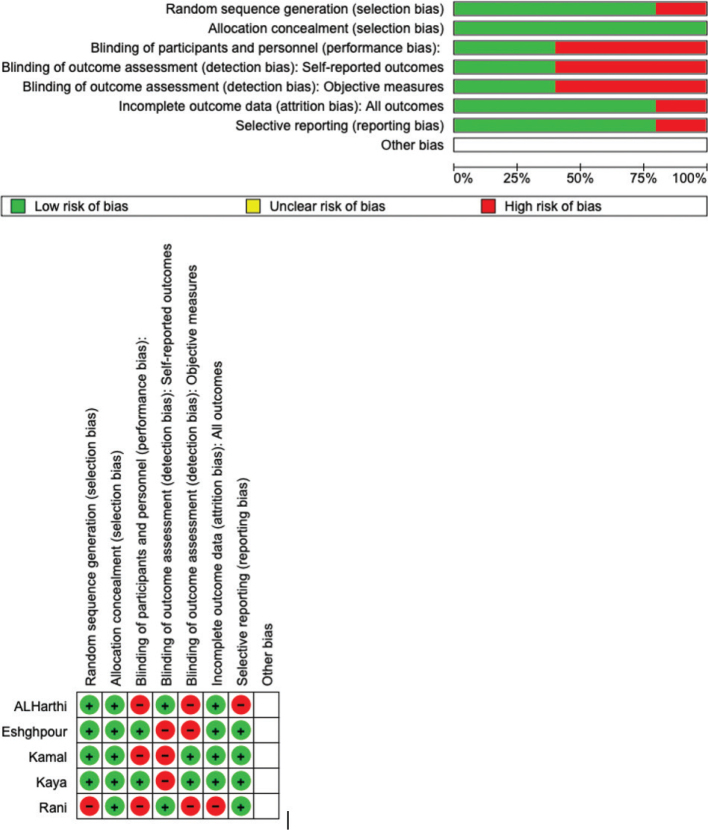
Risk-of-bias domains of included studies.

RoB 2 was used to determine the bias risk, shown in Figure 3. All of the studies ensured a minimal risk of bias about the randomization process. However, bias in the choice of reported outcomes was adequately removed in 100% of the included research but only in 75% of the studies for self-reported outcomes. Though 100% of the studies reported complete outcome data, 75% of them eliminated performance bias. Overall, it was found that all five investigations had a low likelihood of bias.

## Discussion

This review compared different types of treatment for alveolitis. This review and meta-analysis looked at five studies that treated alveolitis with alvogyl and laser application. The meta-analysis showed high heterogeneity and, therefore, unreliable. To make it as homogeneous as possible, we considered painability on a VAS scale 3 days after treatment application [41–56].

Up to 10% of patients undergoing exodontia experience dry sockets connected to excruciating postsurgical pain. Aside from causing excruciating pain, DS can make patients feel unfavorable things like financial burdens, dental anxiety, and fear of OHPs and/or future dental procedures [57, 58]. Alveogyl aids in the healing process for DS sufferers. Accordingly, the authors of the current research hypothesized that DS treatment using Alveogyl in conjunction with PBMT is more effective in lowering SPP than other treatments. Khalighi et al. claim laser applied to tissues triggers biochemical mechanisms that lead to and stimulate tissue healing [59, 60].

Prostatic acid phosphatase, a facilitator of analgesia, is expressed more frequently in tissues due to PBMT, according to Chen et al. [61]. Additionally, it has been suggested that PBMT lessens pain awareness by increasing serotonin synthesis, slowing the conduction of action potentials (APs), and lengthening the latency of the sural and median nerves. Similarly, eugenol-based dressings like Alveogyl stop AP by preventing sodium currents in oral afferent neurons and stabilizing neuronal membranes [61].

DS treatment is palliative since healing happens within 1 to 4 weeks postoperatively. Whatever the technique, cleaning and rinsing the extraction socket are critical to eliminate debris and germs from the denuded bone. Even the patients in our study who got only curettage and irrigation showed symptomatic improvement, albeit slowly, highlighting the significance of this procedure [62]. Curettage and irrigation alone won’t be enough, as evidenced by the statistically significant differences in every parameter investigated between the control and all three treatment groups. The dressing of the extraction socket, which filled the space in the socket, prevented the buildup of debris, relieved pain, cleaned the alveoli, sped up healing, and prevented odor from coming from the empty socket, was equally significant [63]. The active ingredients of the dressings mentioned in studies that have been published have either had antibacterial, analgesic, topical anesthetic, or a combination of these qualities. The majority of cleaning methods have been used for many years. Individual clinicians have their preferences although there is no trustworthy clinical evidence to indicate that one technique has a therapeutic advantage over another, and only anecdotal evidence is available to support their efficacy. Eugenol-containing Alvogyl can also reduce inflammation and have analgesic benefits by preventing prostaglandins from working [64]. Pain management has been done using LLLT. Although the exact mechanism of pain relief is unknown, some studies have indicated that LLLT may help to reduce inflammation by inhibiting the production of potent inflammatory mediators like prostacyclin and cyclooxygenase. The effects of LLLT on wound healing have been linked to increased keratinocyte mobility, early epithelization promotion, more significant fibroblast proliferation, matrix synthesis, and neovascularization enhancement. In the study of Kaya et al., no statistically significant differences in pain scores were found between patients treated with SaliCept (acemannan) and alvogyl (eugenol) throughout a 7-day treatment, proving that acemannan is a successful palliative treatment for DS. But after treatment, the VAS scores dropped with LLLT the fastest [65].

The study of Eshghpour evaluates three groups. The first was treated with algoyl, used after socket irrigation and quickly reduced pain. The pain increased by 4% and 18% after 6 h and between 6 and 12 h of the original examination on the second day. Even though the alvogyl group’s pain deterioration was minimal on day 2, it became statistically significant between the T1-T2 and T0-T2 periods. The second group evaluated the use of 660 nm laser in the second group of this research significantly decreased pain throughout the experiment. While the improvement was most noticeable in the first 6 h following irradiation, there was also a notable increase between 6 and 12 h later, suggesting that the biomodulative effects of LPRL continue for several hours following its administration. After 6 h of intervention on day 1, the VAS dropped from 8.21 to 5.35 degrees, and between 6 and 12 h later, it went from 5.35 to 4.42 degrees. Day 2 VAS improvements were 41% and 47% after 6 h of laser treatment and between 6 and 12 h, respectively. The VAS ratings on day 3 were the lowest, coming close to zero. In the third group, in the third set of this experiment, LLLT was carried out using an infrared laser of 810 nm wavelength. The pain was reduced after the first 6 h of laser application on the first day [66]. On day 2, the VAS improved. On day 3, the comparable figures were 57% and 45%. The overall VAS score reduction on days 1, 2, and 3 was 2.7, 3.1, and 4 degrees, respectively, indicating a rise in the 810 nm diode laser’s effectiveness.

When the study groups’ pain levels were compared, it became clear that the alvogyl group’s pain levels on the first day were considerably lower than those of the other groups at 6 and 12 h following the intervention [67]. This difference was also present at the T0 and T1 time points on day 2 when patients receiving alvogyl reported substantially less pain than those in the other groups. This can be ascribed to the dressing’s ability to fill the empty socket and thereby prevent stimulation of the denuded bone, one of the primary pain-producing factors, from occurring. The dressing also has analgesic and local anesthetic components that can work right away to lessen patients’ pain. But a dressing inside the extraction socket might also be linked to a slower recovery rate. After 12 h of intervention on day 2, the order of pain intensity changed, and the mean VAS decreased considerably more in the red laser group than in the other groups. On day 3, the LPRL group’s considerably reduced pain intensity persisted throughout all treatment intervals compared to the LPIL and alvogyl groups [68]. These findings suggest that the alveogyl reduces pain in patients with dry sockets more quickly than LLLT. Still, the 660 nm laser overcame the alvogyl’s initial advantage after 12 h of intervention on day 2 and at all therapy intervals on day 3. This may be connected to the fact that LLLT can improve the speed and quality of wound healing and reduce discomfort and inflammation.

The current study accelerated the healing process using red and infrared lasers. The alveolar bone inside the extraction socket was given a better chance to mend due to the infrared laser, which has a 2- to 3-cm penetration depth.

According to research by Bjordal et al., the lowest energy density for an infrared laser to have analgesic and anti-inflammatory effects on small and more significant wounds, respectively, is 6 Joules per Square Centimeter (J/cm^2^) and 10 J/cm^2^ [69]. The risk of developing DS significantly decreased using PBM therapy in the first postoperative week. In other words, the PBM therapy group’s chance of developing DS was half that of the sham PBM treatment group (relative risk = 0.52). This meta-analysis showed that in the immediate treatment of pain, alvogyl could quickly help post-alveolitis pain. However, laser treatment is helpful and easy to use to prevent and treat pain and dry alveolitis.

A dry socket, also known as alveolar osteitis, is a postoperative complication that commonly occurs after tooth extraction. It is characterized by severe pain, inflammation, and delayed healing of the extraction socket due to the dislodgement or dissolution of the blood clot. Various treatment modalities have been explored to manage dry sockets effectively. One emerging approach is the use of LLLT. This discussion aims to explore the potential benefits of LLLT in managing dry sockets based on existing scientific literature.

LLLT, also called PBMT, utilizes low-power lasers or light-emitting diodes to stimulate tissue healing and reduce inflammation. The therapeutic effects of LLLT are believed to occur through various mechanisms, including increased cellular metabolism, enhanced blood flow, modulation of inflammatory mediators, and promotion of tissue repair processes.

Several studies have investigated the effectiveness of LLLT in managing dry sockets. One randomized controlled trial (RCT) conducted by Jovanovic et al. [70] evaluated the effects of LLLT on 60 patients with dry sockets. The participants were divided into two groups, one receiving LLLT and the other serving as a control. The results showed that the LLLT group experienced significantly reduced pain, improved healing, and decreased inflammation compared to the control group.

Similarly, another RCT by Elbay et al. [71] assessed the effects of LLLT in treating dry sockets. The study included 37 patients randomly assigned to receive either LLLT or a placebo with primary molar extraction. The LLLT group showed faster pain relief, enhanced wound healing, and reduced edema compared to the placebo group. Moreover, LLLT significantly decreased the need for additional interventions and promoted earlier resolution of dry socket symptoms.

The potential mechanisms underlying the positive effects of LLLT on dry socket management have also been investigated. It has been proposed that LLLT may exert its therapeutic effects by modulating inflammatory mediators such as cytokines by reducing inflammation. Additionally, LLLT has been shown to enhance cellular metabolism and ATP production, facilitating tissue repair and regeneration processes in the extraction socket.

While the existing studies indicate the potential benefits of LLLT in managing dry sockets, some considerations and limitations still need to be addressed. The optimal parameters for LLLT, including wavelength, power density, treatment duration, and frequency, have not been standardized. Additionally, more studies with larger sample sizes and more extended follow-up periods are required to validate the findings and evaluate the long-term outcomes of LLLT in dry socket treatment. Furthermore, the cost-effectiveness and practicality of incorporating LLLT into routine dental practice must be assessed.

LLLT is an adjunctive treatment for dry sockets, with evidence suggesting improved pain relief, faster healing, and reduced inflammation. However, further research is warranted to establish standardized protocols, determine long-term outcomes, and assess the feasibility of integrating LLLT into routine dental care. As our understanding of LLLT mechanisms and their effects on dry sockets expands, it may become a valuable therapeutic option for managing this postoperative complication.

### Limitations of the study

This systematic review includes five articles in the literature. The main weaknesses and, thus, limitations of the study concern:

1. not having considered the type of laser and the power of use
2. not taking into account the age of the population
3. the small sample size
4. the small number of studies.

However, despite this limitation, the results unmistakably show the importance of laser in decreasing pain following DR.

## Conclusions

These results suggest that laser treatment may be an effective alternative to conventional treatments for dry sockets. It is a minimally invasive procedure that can relieve pain and promote healing with fewer side effects. Further research is needed to evaluate the long-term efficacy of laser treatment for dry sockets. Accordingly, the authors of the current research hypothesized that DS treatment used in conjunction with PBMT is more effective in lowering SPP than other treatments.

## Data Availability

The data will be available on reasonable request from the corresponding author.

## References

[1] Ghosh A, Aggarwal VR, Moore R. Aetiology, prevention and management of alveolar osteitis – a scoping review. J Oral Rehabil [Internet]. 2022;49(1):103–13. Available from: https://www.scopus.com/inward/record.uri?eid=2-s2.0-85117703741&doi=10.1111%2fjoor.13268&partnerID=40&md5=a941a4c5836d776b0f2886aa8cfed276 34625985 10.1111/joor.13268

[2] Rosa A, Pujia AM, Arcuri C. Investigation of alveolar osteitis and the effectiveness of laser treatment: a unified Meta-analysis and review of the literature. BMC Oral Health. 2024;24:700. 10.1186/s12903-024-04461-w 38886713 PMC11184811

[3] Miranda M, Gianfreda F, Rosa A, Fiorillo L, Cervino G, Cicciù M, et al. Treatment of oral mucositis using platelet-rich-fibrin: a retrospective study on oncological patients. J Craniofac Surg. 2023 Jul-Aug 01;34(5):1527–9. 10.1097/SCS.0000000000009450 37276338

[4] Winckler K, Rasmussen MU, Laugenborg J, Bukkehave KH, Fischer H, Heitmann BL, et al. Barriers for why pregnant women do not visit a dentist on a regular basis: using group concept mapping methodology. Acta Odontol Scand [Internet]. 2023. Available from: https://www.scopus.com/inward/record.uri?eid=2-s2.0-85177471480&doi=10.1080%2f00016357.2023.2283198&partnerID=40&md5=a58d7215bd505b4428d119c584abf117 10.1080/00016357.2023.2283198 PMC11302643 37982800

[5] Sabel N, Ylander LO, Ståhlberg SE, Robertson A. Dental caries and oral health-related quality of life in Preschoolers–introducing the Swedish version of the early childhood oral health impact scale (ECOHIS). Acta Odontol Scand [Internet]. 2023; Available from: https://www.scopus.com/inward/record.uri?eid=2-s2.0-85178460805&doi=10.1080%2f00016357.2023.2287235&partnerID=40&md5=e26ec76f61663059c4af2581c4022719 10.1080/00016357.2023.2287235 PMC11302645 38032108

[6] Alobaidi F, Heidari E, Sabbah W. Systematic review of longitudinal studies on the association between cluster of health-related behaviors and tooth loss among adults. Acta Odontol Scand [Internet]. 2023. Available from: https://www.scopus.com/inward/record.uri?eid=2-s2.0-85178222083&doi=10.1080%2f00016357.2023.2287718&partnerID=40&md5=29f33fac9fe07a372749a227d6dd21a8 10.1080/00016357.2023.2287718 PMC11302646 38014435

[7] Shmarina E, Stensson M, Jacobsson B. Oral health literacy among migrant mothers in Sweden. A qualitative study. Acta Odontol Scand [Internet]. 2023. Available from: https://www.scopus.com/inward/record.uri?eid=2-s2.0-85179732175&doi=10.1080%2f00016357.2023.2291206&partnerID=40&md5=a202e18182b9dd5a1b435d094244084a 10.1080/00016357.2023.2291206 PMC11302650 38082482

[8] Mariani P, Menditti D, Russo D, Laino L. Evaluation of the effectiveness of tube drain on postoperative discomfort in mandibular third molar surgery: prospective randomized split-mouth study. Acta Odontol Scand. 2023 Oct 3;81(7):528–33. 10.1080/00016357.2023.2205934 37177802

[9] Kusumastiwi PO, Hendrartini J, Dwiprahasto I. The combination of butyl para-aminobenzoate and iodoform as a secondary prevention of dry socket: a systematic review. Open Access Maced J Med Sci [Internet]. 2020;8(F):246–52. Available from: https://www.scopus.com/inward/record.uri?eid=2-s2.0-85097620215&doi=10.3889%2foamjms.2020.5350&partnerID=40&md5=0b08de4888a20330e3303be5200d3612

[10] Minervini G, Nucci L, Lanza A, Femiano F, Contaldo M, Grassia V. Temporomandibular disc displacement with reduction treated with anterior repositioning splint: a 2-year clinical and magnetic resonance imaging (MRI) follow-up. J Biol Regul Homeost Agents. 2020 Jan-Feb;34(1 Suppl. 1):151-160. Dental Supplement. PMID: 32064850. 32064850

[11] Minervini G, Romano A, Petruzzi M, Maio C, Serpico R, Di Stasio D, Lucchese A. Oral-facial-digital syndrome (OFD): 31-year follow-up management and monitoring. J Biol Regul Homeost Agents. 2018 Jan-Feb;32(2 Suppl. 1):127-30. PMID: 29460530. 29460530

[12] Campus G, Diaz-Betancourt M, Cagetti M, Carvalho J, Carvalho T, Cortés-Martinicorena J, et al. Study protocol for an online questionnaire survey on symptoms/signs, protective measures, level of awareness and perception regarding COVID-19 outbreak among dentists. A global survey. Int J Environ Res Public Health. 2020 Aug 3;17(15):5598. 10.3390/ijerph17155598 32756475 PMC7432089

[13] Sabbatini M, Santillo M, Pisani A, Paternò R, Uccello F, Serù R, et al. Inhibition of Ras/ERK1/2 signaling protects against postischemic renal injury. Am J Physiol Renal Physiol. 2006 Jun;290(6):F1408–15. 10.1152/ajprenal.00304.2005 16434573

[14] Krifka S, Petzel C, Bolay C, Hiller KA, Spagnuolo G, Schmalz G, et al. Activation of stress-regulated transcription factors by triethylene glycol dimethacrylate monomer. Biomaterials. 2011 Mar;32(7):1787–95. 10.1016/j.biomaterials.2010.11.031 21145583

[15] Inchingolo AM, Malcangi G, Ferrara I, Patano A, Viapiano F, Netti A, et al. MRONJ Treatment strategies: a systematic review and two case reports. Appl Sci. 2023 Mar 29;13(7):4370. 10.3390/app13074370

[16] Thorat S, Nilesh K. Efficacy of low-level laser therapy in the management of postoperative surgical sequelae after surgical removal of impacted mandibular third molars. Natl J Maxillofac Surg [Internet]. 2022;13(4):S52–6. Available from: https://www.scopus.com/inward/record.uri?eid=2-s2.0-85137760754&doi=10.4103%2fnjms.NJMS_52_20&partnerID=40&md5=bb93a63d9692176945ebad712b6eed4d 36393948 10.4103/njms.NJMS_52_20 PMC9651229

[17] Kazakova R, Tomov G, Vlahova A, Zlatev S, Dimitrova M, Kazakov S, et al. Assessment of healing after diode laser gingivectomy prior to prosthetic procedures. Appl Sci. 2023 Apr 28;13(9):5527. 10.3390/app13095527

[18] Kamal A, Salman B, Ar NH, Samsudin AR. Management of dry socket with low-level laser therapy. Clin Oral Investig [Internet]. 2021;25(3):1029–33. Available from: https://www.scopus.com/inward/record.uri?eid=2-s2.0-85086723103&doi=10.1007%2fs00784-020-03393-3&partnerID=40&md5=2e976c17c0af5e998228ee0c18e7d767 10.1007/s00784-020-03393-3 32562076

[19] Shafaee H, Bardideh E, Nazari MS, Asadi R, Shahidi B, Rangrazi A. The effects of photobiomodulation therapy for treatment of alveolar osteitis (Dry Socket): systematic review and meta-analysis. Photodiagnosis Photodyn Ther [Internet]. 2020;32. Available from: https://www.scopus.com/inward/record.uri?eid=2-s2.0-85091061019&doi=10.1016%2fj.pdpdt.2020.102000&partnerID=40&md5=8fee840a9fb1a000c0556d24ff298e31 10.1016/j.pdpdt.2020.102000 32919077

[20] Konuk B, Senturk M. Three-Dimensional evaluation of the effect of platelet-rich fibrin on edema in lower impacted third molar surgery performed with piezosurgery. Niger J Clin Pract. 2022;25(7):1107. 10.4103/njcp.njcp_1700_21 35859473

[21] Iqbal N, Khalid MU, Janjua OS, Zafar KJ, Usama MM. Assessment of dry socket after mandibular third molar surgery using platelet-rich fibrin – a prospective clinical study. J Coll Phys Surg Pak. 2023 May 1;33(05):504–8. 10.29271/jcpsp.2023.05.504 37190682

[22] Pelivan I, Šeparović I, Vuletić M, Dulčić N, Gabrić D. Radiological and periodontal evaluation of stock and custom CAD/CAM implant abutments – a one-year follow-up study. Prosthesis [Internet]. 2023;5(2):437–52. Available from: https://www.scopus.com/inward/record.uri?eid=2-s2.0-85163779375&doi=10.3390%2fprosthesis5020030&partnerID=40&md5=47c8810172d801c0e5dbcc98eb643954

[23] Choi S, Kang YS, Yeo ISL. Influence of implant–abutment connection biomechanics on biological response: a literature review on interfaces between implants and abutments of titanium and zirconia. Prosthesis [Internet]. 2023;5(2):527–38. Available from: https://www.scopus.com/inward/record.uri?eid=2-s2.0-85163744250&doi=10.3390%2fprosthesis5020036&partnerID=40&md5=aefc7b44f87fbad41c2c5d9ee7c9ca89

[24] Hernández-Ortega MF, Torres-SanMiguel CR, Alcántara-Arreola EA, Paredes-Rojas JC, Cabrera-Rodríguez O, Urriolagoitia-Calderón GM. Numerical assessment of interspinous spacers for lumbar spine. Prosthesis [Internet]. 2023;5(3):939–51. Available from: https://www.scopus.com/inward/record.uri?eid=2-s2.0-85172092372&doi=10.3390%2fprosthesis5030065&partnerID=40&md5=fd371d0b9ff58b6406be7211aeb5f021

[25] Ramadanov N, Marinova-Kichikova P, Hable R, Dimitrov D, Becker R. Comparison of postoperative serum biomarkers after total hip arthroplasty through minimally invasive versus conventional approaches: a systematic review and meta-analysis of randomized controlled trials. Prosthesis [Internet]. 2023;5(3):694–710. Available from: https://www.scopus.com/inward/record.uri?eid=2-s2.0-85172900513&doi=10.3390%2fprosthesis5030049&partnerID=40&md5=f2c6464404446b043c4e90619a4ae403

[26] Rapani A, Berton F, Tramontin A, Turco G, Marchesi G, Di Lenarda R, et al. Surface roughness of enamel and dentin after preparation finishing with rotary burs or piezoelectric instruments. Prosthesis [Internet]. 2023;5(3):711–20. Available from: https://www.scopus.com/inward/record.uri?eid=2-s2.0-85172778398&doi=10.3390%2fprosthesis5030050&partnerID=40&md5=81eb3123240119b35e4932f6d20ab0f4

[27] Al-Shamiri HM, Al-Maweri SA, AlAhmary AW, Aljunayh MS, Aldosari AO, Alqahtani NM, et al. Efficacy of laser therapy for alveolar osteitis: a systematic review of the available evidence. J Evid Based Dental Pract [Internet]. 2022;22(2). Available from: https://www.scopus.com/inward/record.uri?eid=2-s2.0-85127618036&doi=10.1016%2fj.jebdp.2022.101711&partnerID=40&md5=7091e49d611cf6bf74e604a4e379c737 10.1016/j.jebdp.2022.101711 35718430

[28] d’Apuzzo F, Nucci L, Delfino I, Portaccio M, Minervini G, Isola G, Serino I, Camerlingo C, Lepore M. Application of Vibrational Spectroscopies in the Qualitative Analysis of Gingival Crevicular Fluid and Periodontal Ligament during Orthodontic Tooth Movement. J Clin Med. 2021 Apr 1;10(7):1405. 10.3390/jcm10071405 33915746 PMC8036342

[29] Jornet-García A, Sánchez-Pérez A, Planes-Nicolás P, Montoya-Carralero JM, Moya-Villaescusa MJ. Influence of the number of microthreads on marginal bone loss: a five-year retrospective clinical study in humans. Appl Sci. 2023 Mar 20;13(6):3936. 10.3390/app13063936

[30] Khabiri M, Kamgar S, Iranmanesh P, Khademi A, Torabinejad M. Postoperative pain of single-visit endodontic treatment with gutta-percha versus MTA filling: a randomized superiority trial. BMC Oral Health [Internet]. 2023;23(1). Available from: https://www.scopus.com/inward/record.uri?eid=2-s2.0-85180194672&doi=10.1186%2fs12903-023-03372-6&partnerID=40&md5=3b0d9b1c13e65715469a78b60fd6fe98 10.1186/s12903-023-03372-6 PMC10731764 38114967

[31] Németh O, Uhrin E, Girasek E, Boros J, Győrffy Z. The impact of digital healthcare and teledentistry on dentistry in the 21st Century: a survey of Hungarian dentists. BMC Oral Health [Internet]. 2023;23(1). Available from: https://www.scopus.com/inward/record.uri?eid=2-s2.0-85180266059&doi=10.1186%2fs12903-023-03770-w&partnerID=40&md5=9ccbbf0d2f6e2ba61ec004a9cf8e88b4 10.1186/s12903-023-03770-w PMC10731718 38115014

[32] Li X, Zhao D, Xie J, Wen H, Liu C, Li Y, et al. Deep learning for classifying the stages of periodontitis on dental images: a systematic review and meta-analysis. BMC Oral Health [Internet]. 2023;23(1). Available from: https://www.scopus.com/inward/record.uri?eid=2-s2.0-85180191000&doi=10.1186%2fs12903-023-03751-z&partnerID=40&md5=678541a6e14f91d8a6a7e786d4c38725 10.1186/s12903-023-03751-z PMC10729340 38114946

[33] Novrinda H, Azhara CS, Rahardjo A, Ramadhani A, Dong-Hun H. Determinants and inequality of recurrent aphthous stomatitis in an Indonesian population: a cross sectional study. BMC Oral Health [Internet]. 2023;23(1). Available from: https://www.scopus.com/inward/record.uri?eid=2-s2.0-85180218640&doi=10.1186%2fs12903-023-03683-8&partnerID=40&md5=18aa095d88fde96f561e4ca2d903b5b7 10.1186/s12903-023-03683-8 PMC10731680 38114965

[34] Ereifej NS, Oweis YG, Abu-Awwad M. The effect of using denture adhesives on patient satisfaction with complete dentures; a randomized clinical trial. BMC Oral Health [Internet]. 2023;23(1). Available from: https://www.scopus.com/inward/record.uri?eid=2-s2.0-85180186543&doi=10.1186%2fs12903-023-03757-7&partnerID=40&md5=1c238c139f435e08b953727bc2af4819 10.1186/s12903-023-03757-7 PMC10731830 38114958

[35] Ismayılov R, Özgür B. Indications and use of cone beam computed tomography in children and young individuals in a university-based dental hospital. BMC Oral Health [Internet]. 2023;23(1). Available from: https://www.scopus.com/inward/record.uri?eid=2-s2.0-85180500618&doi=10.1186%2fs12903-023-03784-4&partnerID=40&md5=e4a12a0e644dbf1c1fef639494c26f5a 10.1186/s12903-023-03784-4 PMC10740269 38129827

[36] Reda SA, Hussein YF, Riad M. The impact of Bis-GMA free and Bis-GMA containing resin composite as posterior restoration on marginal integrity: a randomized controlled clinical trial. BMC Oral Health [Internet]. 2023;23(1). Available from: https://www.scopus.com/inward/record.uri?eid=2-s2.0-85180240448&doi=10.1186%2fs12903-023-03759-5&partnerID=40&md5=acb1e6a6f7d7b6279b889aeb58a1a79c 10.1186/s12903-023-03759-5 PMC10731870 38114979

[37] ALHarthi SS, Ali D, Alamry NZ, Alshehri MK, Divakar DD, BinShabaib MS. Photobiomodulation for managing ‘Dry Socket’: a randomised controlled trial. Int Dent J. 2023 Apr;73(2):267–73. 10.1016/j.identj.2022.06.002 35803777 PMC10023530

[38] Kaya GŞ, Yapıcı G, Savaş Z, Güngörmüş M. Comparison of Alvogyl, SaliCept Patch, and low-level laser therapy in the management of alveolar osteitis. J Oral Maxillofac Surg. 2011 Jun;69(6):1571–7. 10.1016/j.joms.2010.11.005 21398006

[39] Eshghpour M, Ahrari F, Najjarkar NT, Khajavi MA. Comparison of the effect of low level laser therapy with alvogyl on the management of alveolar osteitis. Med Oral Patol Oral Cir Bucal. 2015;e386–92. 10.4317/medoral.20375 25662557 PMC4464928

[40] Rani A, Mohanty S, Sharma P, Dabas J. Comparative evaluation of Er:Cr:YSGG, diode laser and alvogyl in the management of alveolar osteitis: a prospective randomized clinical study. J Maxillofac Oral Surg. 2016 Sep 11;15(3):349–54. 10.1007/s12663-015-0848-4 27752206 PMC5048323

[41] Garola F, Gilligan G, Panico R, Leonardi N, Piemonte E. Clinical management of alveolar osteitis. A systematic review. Med Oral Patol Oral Cir Bucal [Internet]. 2021;26(6):e691–702. Available from: https://www.scopus.com/inward/record.uri?eid=2-s2.0-85120698804&doi=10.4317%2fmedoral.24256&partnerID=40&md5=88e599ec5869b83e9681303ef0a49703 34704976 10.4317/medoral.24256 PMC8601644

[42] Rokohl AC, Trester M, Naderi P, Loreck N, Zwingelberg S, Bucher F, et al. Dry anophthalmic socket syndrome – morphological alterations in meibomian glands. Eye (Basingstoke) [Internet]. 2021;35(12):3358–66. Available from: https://www.scopus.com/inward/record.uri?eid=2-s2.0-85100750024&doi=10.1038%2fs41433-021-01426-z&partnerID=40&md5=82b04c00bed9371f97d0d802ca8f0a40 10.1038/s41433-021-01426-z PMC8602645 33564141

[43] Di Paola A, Tortora C, Argenziano M, Marrapodi MM, Rossi F. Emerging roles of the iron chelators in inflammation. Int J Mol Sci. 2022 Jul 20;23(14):7977. 10.3390/ijms23147977 35887336 PMC9318075

[44] Marrapodi MM, Mascolo A, di Mauro G, Mondillo G, Pota E, Rossi F. The safety of blinatumomab in pediatric patients with acute lymphoblastic leukemia: a systematic review and meta-analysis. Front Pediatr. 2022 Jul 22;10. 10.3389/fped.2022.929122 PMC9354602 35935358

[45] Inchingolo F, Tatullo M, Marrelli M, Inchingolo AM, Picciariello V, Inchingolo AD, Dipalma G, Vermesan D, Cagiano R. Clinical trial with bromelain in third molar exodontia. Eur Rev Med Pharmacol Sci. 2010 Sep;14(9):771-4. PMID: 21061836. 21061836

[46] Di Cosola M, Cazzolla AP, Charitos IA, Ballini A, Inchingolo F, Santacroce L. Candida albicans and oral carcinogenesis. A brief review. J Fungi. 2021 Jun 12;7(6):476. 10.3390/jof7060476 PMC8231483 34204731

[47] Minervini G, Franco R, Marrapodi MM, Almeida LE, Ronsivalle V, Cicciù M. Prevalence of temporomandibular disorders (TMD) in obesity patients: a systematic review and meta‐analysis. J Oral Rehabil. 2023 Dec 27;50(12):1544–53. 10.1111/joor.13573 37635375

[48] Minervini G, Franco R, Marrapodi MM, Fiorillo L, Cervino G, Cicciù M. The association between parent education level, oral health, and oral-related sleep disturbance. An observational crosssectional study. Eur J Paediatr Dent. 2023 Sep 1;24(3):218-223. 10.23804/ejpd.2023.1910 37668455

[49] Minervini G, Franco R, Marrapodi MM, Di Blasio M, Ronsivalle V, Cicciù M. Children oral health and parents education status: a cross sectional study. BMC Oral Health. 2023 Oct 24;23(1):787. 10.1186/s12903-023-03424-x 37875845 PMC10594879

[50] Franco R, Miranda M, Di Renzo L, De Lorenzo A, Barlattani A, Bollero P. Glanzmann’s thrombastenia: the role of tranexamic acid in oral surgery. Case Rep Dent. 2018 Sep 5;2018:1–4. 10.1155/2018/9370212 PMC6145161 30254767

[51] Franco R, Barlattani A Jr, Perrone MA, Basili M, Miranda M, Costacurta M, Gualtieri P, Pujia A, Merra G, Bollero P. Obesity, bariatric surgery and periodontal disease: a literature update. Eur Rev Med Pharmacol Sci. 2020 May;24(9):5036-5045. 10.26355/eurrev_202005_21196 32432767

[52] Ronsivalle V, Carli E, Lo Giudice A, Lagravère M, Leonardi R, Venezia P. Nasal septum changes in adolescents treated with tooth-borne and bone-borne rapid maxillary expansion: a CBCT retrospective study using skeletal tortuosity ratio and deviation analysis. Children. 2022 Nov 29;9(12):1853. 10.3390/children9121853 36553296 PMC9776818

[53] Lo Giudice A, Ronsivalle V, Santonocito S, Lucchese A, Venezia P, Marzo G, et al. Digital analysis of the occlusal changes and palatal morphology using elastodontic devices. A prospective clinical study including Class II subjects in mixed dentition. Eur J Paediatr Dent. 2022 Dec;23(4):275–80. 10.1186/s12903-023-02731-7 36511914

[54] Lo Russo L, Pierluigi M, Zhurakivska K, Digregorio C, Lo Muzio E, Laino L. Three-dimensional accuracy of surgical guides for static computer-aided implant surgery: a systematic review. Prosthesis. 2023 Sep 4;5(3):809–25. 10.3390/prosthesis5030057

[55] Cervino G, Fiorillo L, Laino L, Herford AS, Lauritano F, Giudice G Lo, et al. Oral health impact profile in celiac patients: analysis of recent findings in a literature review. Gastroenterol Res Pract. 2018 Oct 24;2018:1–9. 10.1155/2018/7848735 PMC6220388 30473707

[56] Laino L, Cicciù M, Fiorillo L, Crimi S, Bianchi A, Amoroso G, et al. Surgical risk on patients with coagulopathies: guidelines on hemophiliac patients for oro-maxillofacial surgery. Int J Environ Res Public Health. 2019 Apr 17;16(8):1386. 10.3390/ijerph16081386 30999657 PMC6518229

[57] Kamal A, Salman B, Razak NHA, Samsudin ABR. A comparative clinical study between concentrated growth factor and low-level laser therapy in the management of dry socket. Eur J Dent [Internet]. 2020;14(4):613–20. Available from: https://www.scopus.com/inward/record.uri?eid=2-s2.0-85092705139&doi=10.1055%2fs-0040-1714765&partnerID=40&md5=863d7553afb680e42661fe43e1755061 32777838 10.1055/s-0040-1714765 PMC7535966

[58] Abe S, Kawano F, Kohge K, Kawaoka T, Ueda K, Hattori-Hara E, et al. Stress analysis in human temporomandibular joint affected by anterior disc displacement during prolonged clenching. J Oral Rehabil. 2013 Apr;40(4):239–46. 10.1111/joor.12036 23398635

[59] Khalighi HR, Anbari F, Beygom Taheri J, Bakhtiari S, Namazi Z, Pouralibaba F. Effect of Low-power Laser on Treatment of Orofacial Pain. J Dent Res Dent Clin Dent Prospects. 2010 Summer;4(3):75-8. 10.5681/joddd.2010.019 22991602 PMC3429981

[60] Franco R, Gianfreda F, Miranda M, Barlattani A, Bollero P. The hemostatic properties of chitosan in oral surgery. Biomed Biotechnol Res J. 2020;4(3):186. 10.4103/bbrj.bbrj_43_20

[61] Chen L, Zhu L, Wang K, Wang W, Mei XP, Liu T, et al. Antinociceptive effect of prostatic acid phosphatase in a rat model of cancer-induced bone pain. Pain Phys. 2013;16(6):533–46. 10.36076/ppj.2013/16/E533 24284839

[62] Coulthard P, Bailey E, Esposito M, Furness S, Renton TF, Worthington HV. Surgical techniques for the removal of mandibular wisdom teeth. Cochrane Database Syst Rev. 2014; (7):CD004345. 10.1002/14651858.CD004345.pub2 25069437

[63] Brignardello-Petersen R, Carrasco-Labra A, Araya I, Yanine N, Beyene J, Shah PS. Is adjuvant laser therapy effective for preventing pain, swelling, and trismus after surgical removal of impacted mandibular third molars? A systematic review and meta-analysis. J Oral Maxillofac Surg. 2012 Aug;70(8):1789–801. 10.1016/j.joms.2012.01.008 22398186

[64] Ferrante M, Petrini M, Trentini P, Perfetti G, Spoto G. Effect of low-level laser therapy after extraction of impacted lower third molars. Lasers Med Sci. 2013 May 28;28(3):845–9. 10.1007/s10103-012-1174-4 22843310

[65] Aras MH, Güngörmüş M. Placebo-controlled randomized clinical trial of the effect two different low-level laser therapies (LLLT) – intraoral and extraoral – on trismus and facial swelling following surgical extraction of the lower third molar. Lasers Med Sci. 2010 Sep 31;25(5):641–5. 10.1007/s10103-009-0684-1 19484402

[66] La Rosa GRM, Marcianò A, Priolo CY, Peditto M, Pedullà E, Bianchi A. Effectiveness of the platelet-rich fibrin in the control of pain associated with alveolar osteitis: a scoping review. Clin Oral Investig. 2023; 27(7):3321-30. 10.1007/s00784-023-05012-3 PMC10329583 37014504

[67] Zhu J, Zhang S, Yuan X, He T, Liu H, Wang J, et al. Effect of platelet-rich fibrin on the control of alveolar osteitis, pain, trismus, soft tissue healing, and swelling following mandibular third molar surgery: an updated systematic review and meta-analysis. Int J Oral Maxillofac Surg. 2021 Mar;50(3):398–406. 10.1016/j.ijom.2020.08.014 32950350

[68] Eshghpour M, Danaeifar N, Kermani H, Nejat AH. Does intra-alveolar application of chlorhexidine gel in combination with platelet-rich fibrin have an advantage over application of platelet-rich fibrin in decreasing alveolar osteitis after mandibular third molar surgery? A double-blinded randomized clinical trial. J Oral Maxillofac Surg. 2018 May;76(5):939.e1–e7. 10.1016/j.joms.2017.12.009 29316445

[69] Bjordal JM, Johnson MI, Iversen V, Aimbire F, Lopes-Martins RAB. Low-level laser therapy in acute pain: a systematic review of possible mechanisms of action and clinical effects in randomized placebo-controlled trials. Photomed Laser Surg. 2006 Apr;24(2):158–68. 10.1089/pho.2006.24.158 16706694

[70] Jovanovic G, Buric N, Krunic N, Tijanic M, Stojanovic S. Assessment of the effectiveness of low level laser in the treatment of alveolar osteitis. Vojnosanit Pregl. 2011;68(6):506–10. 10.2298/VSP1106506J 21818918

[71] Elbay ÜŞ, Tak Ö, Elbay M, Uğurluel C, Kaya C. Efficacy of low-level laser therapy in the management of postoperative pain in children after primary teeth extraction: a randomized clinical trial. Photomed Laser Surg. 2016 Apr;34(4):171–7. 10.1089/pho.2015.4045 26977740

